# Plaster of Paris–Short History of Casting and Injured Limb Immobilzation

**DOI:** 10.2174/1874325001711010291

**Published:** 2017-04-17

**Authors:** B. Szostakowski, P. Smitham, W.S. Khan

**Affiliations:** University College London Institute of Orthopaedics and Musculoskeletal Science, Royal National Orthopaedic Hospital, Stanmore, HA7 4LP, London, UK

**Keywords:** Immobilisation, Plaster of Paris, Non-operative management, Fractures, Complications

## Abstract

Various materials have been used since ancient times to help immobilise fractures. In this review, we discuss the history and developments of these materials as well as plaster of Paris. There has been a recent trend away from non-operative management of fractures, and skills in the use of plaster of Paris are declining. For the successful treatment of patients, it is important to appreciate how plaster works, how it should be used, and what can go wrong. In this review, we also discuss principles of applications and complications of plaster of Paris.

## HISTORY OF INJURED LIMB IMMOBILIZATION

Immobilization of injured limbs has been performed for thousands of years. Before contemporary casting materials became widely used, people used a variety of materials to form rigid casts. Over the centuries immobilization has evolved from using simple wooden splints and rags to plaster of Paris, fibre and soft casts.

The earliest examples of the active management of fractures in humans were discovered at Naga-ed-Der in 1903 during the Hearst Egyptian Expedition of the University of California lead by Dr. GA Reisner [1, 2]. In a paper published in the British Medical Journal in 1908, Mr. G. Elliot-Smith describes two sets of splints that were found during excavations of tombs from the fifth dynasty (2494-2345 BC) [3]. One of the earliest descriptions of casting material was by Hippocrates in 350 BC. He wrote about wrapping injured limbs in bandages soaked in wax and resin [1, 4]. According to the earliest known surgical text *The Edwin Smith Papyrus* (copied circa 1600 BC), the Egyptians were using self setting bandages, probably derived from those used by the embalmers [1, 4]. Later descriptions of casting came from the Arab physician Rhazes Athuriscus [1]. El Zahrawi (960-1013 AD), a surgeon born near Córdoba in Spain, described the use of both clay gum mixtures and flour and egg white as casting materials [1]. Starch based casts appear to have been the standard treatment with only minor changes until the beginning of the 19^th^ century with only a few minor changes [5].

Further advances in the choice of materials were made during the wars. In the 18^th^ century, Henri François Le Dran, who practiced surgery at Hôpital de la Charité in Paris and was a surgeon in Germany Army and consulting surgeon to the camps and armies of King Louis XV, used to soak his bandages with egg white, vinegar and clay powder or plaster [1, 6]. A modification to the materials used was introduced by the father of modern military surgery, Baron Dominique Jean Larrey, a French surgeon in Napoleon’s army. He was surgeon in chief from 1797 till the Battle of Waterloo in 1815 [7]. Larrey’s modification was adopted from Don Eugenio de la Penna who bandaged the fracture with linen that had first been moistened with Camphor spirit, egg whites and lead-acetate. Unfortunately these were not used on a large scale due to costs [8]. Baron Louis Joseph G Seutin (1793-1862) was a belgian professor and surgeon in chief at the Universite Libre de Bruxelles. As a chief doctor of the Belgian Army he fought in at Waterloo. He became famous for inventing starch bandages known as “La Bandage Immobile” or “L’Appareil Amidonnee” that consisted of strips of linen or bandages and carton splints, soaked in starch and wrapped around the limb [1, 8, 9].

Seutin’s method was popular in England by Joseph Samson Gamgee, the Birmingham surgeon who amongst other things invented Gamgee tissue. In the first half of the 19^th^ century, it was not popular to reduce fractures until the swelling of the soft tissue decreased. Following Seutin’s rules Gamgee insisted on immediate reduction and application of the starched apparatus, and registered spectacular success [10, 11].

### ORIGINS OF PLASTER OF PARIS

Plaster of Paris is produced by removing the impurities from the mined gypsum and then heating it under controlled conditions to reduce the amount of water of crystallization [12]. Plaster of Paris was well known as a building material for many centuries before it was introduced as casting material. Egyptians as well as Romans used it for plastering walls however not more is known on plaster use after the end of Roman occupation. In modern day England, it was widely excavated in Roman coffins discovered in York, and on the walls in the military barracks of the Second Augustian Legion excavated at Caerleon in Monmouthshire [4]. In mediaeval times gypsum was used only for alabaster statuary [4]. There are various accounts describing the origin for the name plaster of Paris. One account mentions King Henry III who visited Paris in 1254 and was so impressed by fine white walls that he introduced similar plastering in England where it became known as plaster of Paris.

The first use of plaster of Paris as a cast for injured limbs took place through a technique known as *plâtre coulé* that became popular in Europe at the beginning of 19^th^ century. This technique involved pouring plaster of Paris around injured limbs encased in a wooden construct. Due to the weight of the construct, the patient was largely confined to bed during the period of fracture healing. This disadvantage was highlighted by Seutin, but this remained a relatively popular technique in Europe with some surgeons using it for lower limbs only and some using it for both upper and lower. Starched and albuminated bandages were also used as a casting method [1].

In 1839, Lafargue of St. Emilion used fresh warm starch paste mixed with plaster of Paris powder applied to layers of linen strips. That dressing had the advantage of hardening much quicker, reducing setting time down to six hours [8, 13]. The Dutch military surgeon Anthonius Mathijsen while working at the military hospital in Haarlem discovered that bandages soaked in water and plaster of Paris were becoming hard within minutes providing sufficient casting for injured limbs. He published his monograph in 1852 in a medical magazine called *Repertorium*. His plaster bandage was based on the principles of Seutin, who 10 years earlier introduced starched bandages known as *bandage amidonnee* [1, 8]. In his paper entitled “*New Method for Application of Plaster-of-Paris Bandage*”, Mathijsen highlighted many disadvantages of Seutin’s dressings including lack of self-adjustment to the changing conditions of the limb, long duration of days needed for the casing to become sufficiently solid, carton splints shrinking and becoming shorter when they dried off adversely affecting fractures, and in cases of suppuration or with small children urinating, dressing becoming soft and loosen [8]. Mathijsen’s bandages consisted of strips of coarse cotton cloth with finely powdered plaster rubbed in. This method of preparation was used until 1950.

Nikolay Ivanovich Pirogov, a head of the department of surgery at the St Petersburg Medico-Surgical Academy and a Russian army surgeon during the Crimean War, conceived his idea to use plaster splints around 1852 while observing the work of a sculptor who used strips of linen soaked in liquid plaster to make models. Pirogov used coarse cloth, either in large pieces or in strips that were immersed in a liquid mixture of plaster of Paris immediately before applying them to limbs protected by stockings and cotton pads. Based on his Crimean experience, Pirogov believed that all patients with fractures due to missile wounds should not be evacuated from the forward dressing stations until the limb had been immobilized in a proper dressing of plaster of Paris [14]. After the war he refined his method by cutting coarse sail cloth to a defined pattern shaped to fit a part of body and soaking it in plaster before application [1, 4].

Use of plaster of Paris bandages for fracture casts became widespread after Mathijsen’s death and replaced most other forms of splintage [1]. Early plaster bandages used at hospitals were made by nursing staff. They were usually freshly made from plaster powder kept in air tight containers that was applied on to the woven bandage or strips of cloths. Care was required while soaking dry bandage in water to prevent the plaster coming off the bandages and dissolving in water. In the early 1930’s, the first commercially manufactured bandages were available in Germany. They were made by spreading plaster mixed with minute quantities of volatile liquids on soft cloth.

### APPLICATION OF PLASTER OF PARIS

Plaster of Paris (2CaSO_4_.H_2_O) is calcium sulphate with water. It is prepared by heating gypsum (CaSO_4_.2H_2_O) at 120°C to allow partial dehydration. When mixed with water, it gives out heat and quickly sets to a hard porous mass within 5 to 15 minutes. The first step is called the setting stage with a slight expansion in volume. The second stage is the hardening stage.

Properties of plaster of Paris bandages have not greatly changed since their first use in the 19^th^ century. Plaster is still widely popular, it is cheap, non-irritant and easy to apply. As quoted by AJ Steele in his article from 1893 on the use of plaster of Paris in orthopaedics, *“The property of rapidly hardening when once wet, gives to plaster its value. Additionally it has merit in its cheapnesss and convenience; it is ever ready, is easily prepared, and simple in its application”* [15]. In 1906, Meisenbach published a 24 pages study on plaster of Paris bandages in the American Journal of Orthopaedic Surgery. He outlined the four essential properties of plaster dressings to include strength, quick set, light weight and ventilation, summarizing that ideal plaster dressing should be thin and strong [16].

Plaster can be used not only for treatment of fractured bones but also supports sprained ligaments, and inflamed and infected soft tissues. It usually sets in few minutes, but needs between 36-72 hours to completely dry. Leg plasters are able to bear weight after 48 hours. Completely dry casts when tapped with knuckles will sound crisp and clear whereas wet casts emit a dull sound. Cast should only be dried by natural methods. No artificially generated heat is recommended. Despite its frequent use, allergic reactions to plaster of Paris are extremely uncommon. There are only a few cases of allergic contact dermatitis from benzalkonium chloride described in the literature [17]; benzalkonium chloride has been used as an additive in certain brands of plaster of Paris since the 1970’s in order to improve its binding properties [18]. When plaster of Paris dries off it becomes porous which helps to maintain patient’s skin free from moisture. It is radiolucent which makes X-ray examination possible. The strength of the plaster cast is determined by the quality of plaster, water to gypsum ratio, product age and storage conditions [19].

The success of non-operative treatment of fractures relies on a clear understanding of fracture healing and the proper use of stabilizing techniques. Non-operative management of fractures has been declining in recent years due to significant advances in operative technology and greater patient expectations of an early return to activity. Younger surgeons are not as familiar with non-operative treatment of fractures with a plaster cast as their predecessors. This is due to a lack of experience in application of plaster casts and the subsequent management. Plaster of Paris is unique and still remains the favoured casting material in many countries. It is cheap, non-toxic, and can easily be moulded to the desired shapes and contours of the body. Skin irritation and allergy is extremely rare.

Application of plaster of Paris requires good knowledge of anatomy and pathology that we are aiming to treat. It has to be applied with a great care that is also need in its supervision afterwards. The perfect plaster dressing must retain the limb under all conditions in the desired position with complete comfort. It must be strong yet light, effective in use but easily removed when no longer required [20].

Prior to casting, any skin lesions or soft tissue injuries must be carefully noted. It is important to observe and document neurovascular status of the extremity, and this needs to be repeated following application of plaster. Patients with neuropathy or neurologic deficits are at greater risk for skin problems with abnormal sensation under the plaster. It is crucial that plaster bandages are rolled on to the limb and not pulled. Figure of eight turns, creases and ridges have to be avoided. Rubbing and massaging plaster bandages during application helps to bond layers together creating stronger and lighter casts. Plaster bandages should be soaked in tepid or slightly warm water. Plaster sets quicker with warm water compared with cold water. The faster the material sets the greater heat produces and the greater the risk of burns [21]. Fast setting plasters have increased risk of thermal injury [12, 16]. There is a risk if casts are allowed to dry resting on pillow. Temperature elevations could be related to the plaster being dipped too briefly and the water being squeezed too aggressively out of the plaster. The water helps release heat, and if there is not enough, the plaster gets hotter.

Lavalette and Ganaway proposed that pre-existing plaster residue in the water might also play a role in elevating cast temperature by maintaining the peak temperature for a longer period, therefore water should be clean [12, 14]. Water temperature of 32 degrees Celcius can be high enough to cause burns. Moritz and Henriques showed that 6 hours at 44 degrees Celcius can cause a third degree burn [12, 14].

A fiberglass cast is a newer synthetic alternative to plaster of Paris. Fiberglass cast is a lightweight and extremely strong material. Fiberglass, also called glass-reinforced plastic (GRP) or glass fiber reinforced plastic (GFRP) is a fiber reinforced polymer made of a plastic matrix reinforced by fine glass. As compared to traditional plaster of Paris cast, it is light in weight and more durable. It is three times stronger and but is only one third in weight. Fiberglass cast is a lightweight and extremely strong material. Fiberglass cast is used for fracture management but is not applied in the acute settings because it is less accommodating to swelling and does not allow moulding.

### COMPLICATIONS ASSOCIATED WITH SPLINTING AND CASTING

There are risks associated with plaster cast immobilization and patient has to be made aware of these. Patients with known diabetes or sensory impairment due to spinal cord injury are those who need particular attention at the time of plaster application and later. Below we discuss some common complications.

### 1 Deep Vein Thrombosis (DVT)

Prolonged lower limb immobilization in plaster carries the risk of deep vein thrombosis (DVT) that the patient has to be made aware of. Two independent studies found that adults treated with a lower extremity cast for an average of 3 weeks had an incidence of DVT between 15% and 36%. Low molecular weight heparin did not significantly reduce the risk of developing DVT [22-24]. Although these are more common in the lower limbs, these have also been described in upper limb immobilisation.

### 2 Compartment Syndrome

One of the most serious complications to be considered is compartment syndrome. This is a condition in which increased pressure within a limited space compromises the circulation and function of the tissues within that space. Compartment syndrome may lead to fatal complications including major loss of limb function and even death [25, 26] and are more common in lower leg and forearm fractures.

### 3 Soft Tissue Swelling

Soft tissue swelling associated with the fractured limb will usually subside within 48 hours from the injury leaving the cast loose. This may lead to displacement of well positioned or reduced fracture, and the reapplication of a new well-fitted cast may be needed. This is more likely to be an issue with unstable fractures. This is more noticeable in lower limb injuries where after education and elevation, swelling can reduce significantly. It is vital to ensure sufficient padding with swelling to prevent complications.

### 4 Pressure Sores

Plaster pressure sores can occur as a result of poor plastering technique associated with inadequate skeletal protection or failure to trim the extremities of the cast correctly. Foreign bodies especially with young children can be easily misplaced in the cast and exert pressure on the skin that can lead to a break in the skin. Every patient should be warned about dangers of scratching beneath the cast with different sharp implements as this can cause infection. Cutting windows in plasters and leaving them unprotected may lead to oedema developing within the window area that will lead to soreness of the skin at the margins. Bivalving casts can be considered as an alternative to enable inspection.

### 5 Venous Congestion

Swelling or blue discoloration of the extremities suggests impaired venous return due to tightness of the plaster. The blue discoloration of venous congestion must be differentiated from bruising.

There are a number of other complications that relate to long periods of immobilization and include joint stiffness, muscle atrophy, cartilage degradation, ligament weakening, and osteoporosis. Some risks can be minimized with correct casting technique [23]. It is important to make patients aware of what can potentially go wrong with a plaster cast.

Our review article shows that plaster of Paris has stood the test of time and is still commonly used. Although there have been developments with the use of the lighter, stronger and more durable synthetic fiberglass of Paris, plaster of Paris is still more widely used as it can be used in the acute setting and allows moulding. It is important to appreciate the complications and how these can be avoided to ensure we continue to use it safely.
